# Effect of Edible Coating Made from Arrowroot Flour and Kaffir Lime Leaf Essential Oil on the Quality Changes of Pork Sausage under Prolonged Refrigerated Storage

**DOI:** 10.3390/foods12193691

**Published:** 2023-10-08

**Authors:** Karthikeyan Venkatachalam, Supaporn Ieamkheng, Paramee Noonim, Somwang Lekjing

**Affiliations:** 1 Faculty of Innovative Agriculture and Establishment Project, Prince of Songkla University, Surat Thani Campus, Makham Tia, Mueang, Surat Thani 84000, Thailand or drkarthikeyan.v@outlook.com (K.V.); paramee.n@psu.ac.th (P.N.); 2 Division of Plant Production Technology, Faculty of Agriculture and National Resources, Rajamangala University of Technology Tawan-ok, Bang Pra, Si Racha, Chonburi 20110, Thailand; supaporn_ie@rmutto.ac.th

**Keywords:** arrowroot tuber flour, kaffir lime leaves essential oil, edible coating, pork sausages, refrigerated storage, quality analysis

## Abstract

Edible coatings are pivotal in enhancing the quality of processed meat products, acting as barriers to environmental and microbial influences by adhering directly to the food surface. Arrowroot flour, a widely produced edible tuber in Thailand, is uncharted in terms of its capability and effectiveness as an edible coating on food materials. This study aims to elucidate the composition and spectral properties of arrowroot tuber flour (ATF) to discern its viability as an edible coating for pork sausages. ATF exhibited a composition predominantly featuring carbohydrates (74.78%), moisture (9.59%), and protein (8.89%), underlining its appropriateness as an edible coating. Rapid visco amylograph revealed ATF’s significant pasting capability. This study incorporated kaffir lime leaves essential oil (KEO) into the ATF coating in diverse concentrations (0–3%). Fourier-transform Infrared spectroscopy illuminated characteristic peaks and bands, showing observable shifts with the integration of KEO, yet the majority of peak placements remained essentially unchanged. The microstructure of the coatings maintained its homogeneity at heightened KEO concentrations, reflecting compatibility with ATF. The efficacy of the ATF-KEO coatings was evaluated on pork sausages, using uncoated samples as controls. While color modifications were evident, coated sausages maintained consistent moisture content, water activity, and pH levels throughout the storage duration. The coated samples also manifested enhanced textural attributes and a decline in lipid oxidation, as evidenced by reduced TBARS levels compared to controls. A subsequent microbial examination corroborated the inhibitory capacity of the ATF-KEO coatings on the microbial proliferation in pork sausages, encapsulating Total Viable Count (TVC), psychrotrophic bacteria, and lactic acid bacteria. In conclusion, the findings substantiate the promising application of ATF, especially in synergy with KEO, as a proficient edible coating for meat products. This combination aids in preserving color and texture, impeding microbial advancement, and moderating lipid oxidation, thereby contributing to the overall quality and safety of the products.

## 1. Introduction

Meat is a crucial part of modern diets, recognized for its rich deposits of protein, vitamins, minerals, and other essential nutrients. It is a prominent source of necessary amino acids and essential fatty acids, including linoleic, linolenic, and oleic acids [1]. According to 2018 figures, meat consumption worldwide was recorded at roughly 346.14 million tons. Projections suggest a potential increase in meat production by 44%, estimated to reach around 453 million tons by 2030. Despite its nutritional importance, meat and meat-based products are susceptible to microbial infection and oxidative degradation [2]. Oxidative reactions can degrade the quality of meat products and pose potential health hazards to consumers [3]. Additionally, the presence of harmful microorganisms can compromise food safety. Among a variety of processed meats, pork sausages are a globally cherished culinary tradition. The preparation typically involves carefully grinding fresh pork meat and fat, and then blending them with a mix of seasonings and spices. Standard spices include pepper powder and hot paprika, while flavor enhancers often used are salt, rice wine, sugar, monosodium glutamate, and ginger. Sausage production involves stuffing this mixture into natural casings, derived from hygienically sanitized pig intestines, followed by thorough cooking [4]. However, the shelf life of cooked pork sausage products tends to be relatively short. This can be ascribed to factors such as inadequate color retention, the onset of rancidity, and other deteriorative elements affecting their quality [5]. To mitigate these issues, it is essential to envelop these products in a polymeric film or coating. This strategy helps protect the sausages from unfavorable conditions, thereby inhibiting premature spoilage [6].

Materials used for packaging play a pivotal role beyond mere containment and protection of food. They also increase the ease of use and effective communication. The packaging industry has progressively become an integral part of the global economic structure, contributing to 2% of the Gross National Product (GNP) in advanced nations [7]. The significance of packaging in the food sector cannot be understated. It ensures the preservation of product quality, freshness, and safety, serving as a barrier against potential microbial attacks, oxidative damage, adulteration, and premature decay [8]. Despite its widespread use in food packaging, plastic is causing ongoing environmental concerns due to its non-degradable characteristics and enduring resistance. As a consequence, there is a growing shift towards the use of biopolymers, sourced from sustainable agricultural leftovers and residuals from the food industry, as a viable substitute [9]. These alternatives are intended to fulfill environmental sustainability needs and address consumer preferences for products that are more in tune with nature. Edible coatings, serving as a defensive barrier for food items, can efficiently defend against deterioration caused by factors such as microbial intrusion, gas permeation, and moisture transfer [10]. Functional ingredients like antioxidants and antimicrobial agents can be incorporated into coatings, potentially allowing a gradual release of additives onto the product surface and thereby enhancing its barrier properties [11]. The emergence of edible films and coatings, celebrated for their edibility, degradability, and harmless impact on both the human body and the environment, represents a significant advancement in sustainable packaging [12]. Among biopolymers, those based on polysaccharides have been extensively researched due to their advantages such as low cost, biocompatibility, biodegradability, and non-toxicity [13].

Arrowroot (*Maranta arundinacea*), a perennial tropical tuber crop native to the tropical forests of South America, belongs to the Marantaceae family [14]. This species presents a promising source of starch due to its high amylose content, around 35%, outcompeting other sources like corn starch (28–33%), wheat starch (30–32%), potato (18–20%), and cassava starch (16–19%), all of which are crucial for film production [15]. Despite their high starch content, arrowroots are often overlooked as a significant starch source due to their low socioeconomic importance in many countries; hence they are not considered a priority raw material [16]. Nonetheless, the high amylose content in arrowroots facilitates the formation of continuous matrices, resulting in the formation of biopolymers with excellent functional properties [17]. Arrowroot starch (ATS) is known for its excellent characteristics, such as high digestibility and gelling ability. Abdillah and Charles [18] reported that an edible polymer made from arrowroot flour (ATF) demonstrated transparent properties along with strong physical and mechanical attributes. Utami Hatmi et al. [19] highlighted the high digestibility nature of ATF, which can be considered advantageous for its application in edible coatings. Nevertheless, despite these positive characteristics, normally the polysaccharide-based edible films and coatings, which include ATF, tend to exhibit low resistance to water vapor and moisture due to their inherent hydrophilic nature. Therefore, the combination of polysaccharides and lipids often leads to the formation of emulsion films or coatings, which feature a hydrophilic matrix and a hydrophobic dispersed phase [20]. There is an emerging technology that concurrently integrates polysaccharide-based edible polymers and essential oils into food coatings. This promising technology aims to inhibit microbial growth, retard surface oxidation, enhance sensory quality, and extend the shelf life of food samples [21].

The kaffir lime (*Citrus hystrix* DC.) is a versatile citrus plant with considerable potential for essential oil production, in addition to its primary role as a condiment. Varieties of condiments from the citrus family abound, including the leaf of the kaffir lime [22]. The kaffir lime can be readily differentiated from other citrus species due to its bifoliate leaves. The commercial value of kaffir lime essential oil (KEO) is derived from its diverse applications, including those in the fragrance, cosmetics, and pharmaceutical industries [23]. In the realm of pharmaceuticals, KEO is utilized as an antioxidant and antibacterial agent. The potency of KEO’s antioxidant and antibacterial activities is directly affected by the composition of the oil [24]. Thirty-eight constituents have been identified in the KEO, comprising 89% of the total oil. KEO is rich in monoterpenes (87%) with ß-pinene being the major component (10%) and a relatively low limonene content (4.7%). The essential oil of *Citrus hystrix* is characterized by a high content of terpinen-4-ol (13.0%), α-terpineol (7.6%), 1,8-cineole (6.4%), and citronellol (6.0%) [25,26]. Kaffir lime essential oil (KEO) has been widely used in edible polymers to preserve various types of foods from spoilage. However, the release of essential oils (EOs) from polymeric film into the food is a complex process. This process is mainly affected by the properties of the polymer matrix, the nature of the antioxidant, and the characteristics of the food.

However, as of now, there are no resources or strategies for using ATF and KEO to create an edible coating that can preserve pork sausages. This study seizes this opportunity to fill this gap by examining the ATF-KEO coating, its properties, and its effectiveness in extending the shelf life of pork sausages without altering their natural characteristics.

## 2. Materials and Methods

### 2.1. Chemicals and Reagents

The essential oil derived from kaffir lime leaves (*Citrus hystrix*) utilized in this study was procured from Thai China Flavors and Fragrances Industry Co., Ltd., located in Bangkok, Thailand. This study employed all the chemicals of analytical grade, which included chloroform, methanol, trichloroacetic acid, potassium iodide, anhydrous sodium sulfate, hydrochloric acid, sodium hydroxide, sodium thiosulfate, thiobarbituric acid, and potassium dihydrogen phosphate, which were purchased from Merck, Darmstadt, Germany and 1,1,3,3-tetramethoxypropane was obtained from Sigma-Aldrich, St. Louis, MO, USA. Additionally, the chemicals used in coating emulsion, including Glycerol and Tween 80, were obtained from J. T. Baker, NJ, USA. All the culture mediums necessary for microbial analysis were bought from HiMedia, Mumbai, India.

### 2.2. Preparation of ATF

Arrowroot tubers (*Maranta arundinacea* L.) that were 12 months old were purchased from a contracted farmer in Sai Yok district, Kanchanaburi province, western Thailand. Arrowroot tuber flour (ATF) was extracted according to the method outlined by Soem [27]. The collected arrowroot tubers were washed with distilled water and then dried at room temperature overnight. They were subsequently ground in a blender (WF-20B, Thaigrinder, Thailand), with distilled water added at a ratio of 1:2. The mixture was filtered through a cloth filter, allowing the ATF to settle. This process was repeated two more times. Afterward, the water containing ATF was drained, and the remaining powder was dried in a hot air oven (Binder, model FD 115, Tuttlingen, Germany) at 60 °C for 4 h. It was then ground in a hammer mill (Retsch, model ZM 1, Burladingen, Germany) and sieved through a 100 mesh screen. The collected ATF was stored in a polyethylene-based Ziplock bag and kept in the refrigerator for further analysis. The proximate compositions, including moisture content, fat content, protein content, fiber content, ash content, and carbohydrate content, as well as the pasting profiles, were analyzed following the AOAC methods.

### 2.3. Preparation of ATF-KEO Coating Emulsion

The ATF coating was prepared in accordance with the method described by de Oliveira Filho et al. [28], with slight modifications. A 1% (*w*/*v*) ATF solution was dispersed in distilled water and heated at 85 ± 2 °C on a magnetic stirrer with a hot plate, and continuously stirred for 10 min. Afterward, 0.5% (*w*/*v*) glycerol and 0.2% (*w*/*v*) tween 80 were added to the coating emulsion mixture, which was then continuously stirred for another 10 min. Once the heat treatment was complete, the coating solution temperature was lowered to 27 ± 2 °C for the coating of the pork sausage (as outlined in Section 2.4). For the preparation of the ATF-KEO coating, the procedure was identical, except that 1, 2, and 3 wt% of KEO was incorporated into the coating emulsion solution after the addition of glycerol and tween 80. The remaining procedure was then carried out as described above. To examine and characterize the ATF-KEO coating, the coating emulsion was cast onto a Petri dish (10 cm in diameter) and then dried in a hot air oven (WTB Binder, FD 56, Germany) at a set temperature of 50 °C for 5–8 h. The dried films were subsequently carefully peeled off from the Petri dish. The dried films were then assessed using a scanning electron microscope (SEM) [29] and Fourier-transform infrared spectroscopy (FTIR) [30].

### 2.4. Coating Pork Sausage and Storage

Ready-to-eat cooked pork sausages were sourced from Betagro Public Company Limited, based in Bangkok, Thailand. The commercial sausages had a shelf life of 30 days. At the time of conducting this experiment, the sausages were 2 days post manufacture. All the sausages were wiped to remove any moisture from the surface and then proceeded to be dipped in the coating solution. The samples were dipped in the coating solution (control (no coating), ATF, ATF-KEO 1–3%) for 3 min. Afterward, the samples were dried for 60 min under the laminar chamber (Human Lab, model CB-180-B-A2-D, Daejeon, Republic of Korea) with continuous airflow conditions and packed into PE ziplock bags, with each bag holding 100 g of sausages. Subsequently, the packed sausages were kept in a refrigerator at 4 ± 2 °C for a storage period of up to 30 days. At regular intervals of 5 days, the samples were measured for various qualities, as described in Section 2.5. The analysis for Day 0 of the sampling was conducted after 12 h of refrigerated storage.

### 2.5. Quality Analysis

#### 2.5.1. Determination of Color Characteristics

To measure the surface color of each sample type, a Hunter Lab color analyzer (HunterLab, model MiniScan EZ, Reston, VA, USA) calibrated with a standard white plate was employed. The color analysis involved recording the L*, a*, and b* values. The L* value indicates lightness, with a value of 0 for black and 100 for white. On the other hand, the a* value represents the red/green scale, where positive values indicate red and negative values indicate green. Lastly, the b* value reflects the yellow/blue scale, with positive values representing yellow and negative values representing blue. The measurement was conducted under *C (D65) illuminant conditions, with a standard observer angle of 10°. The area measured on each sample was 1.25 inches in diameter. Furthermore, a numerical total color difference (ΔE) was estimated on the pork sausages during storage by following the equation proposed by MacDougall [31]:

$$\Delta E={\left[\;{\left(L-L_{\mathrm{ref}}\right)}^{2}+{\left(a-a_{\mathrm{ref}}\right)}^{2}+{\left(b-b_{\mathrm{ref}}\right)}^{2}\right]}^{1/2}$$

The control sausages that have no coating were used as values for L_ref_, a_ref_, and b_ref_.

#### 2.5.2. Determination of pH

To assess the pH levels [32], 10 g of sausage samples were carefully homogenized using a high-shear homogenizer (IKA, model T25 digital ULTRA-TURRAX, Staufen, Germany) combined with 20 mL of distilled water. The homogenizer was set to operate at a speed of 12,000 rotations per minute for a total time of 60 s. Following this process, the homogenized sample was collected, and the pH values were determined using a pH meter (Mettler Toledo, model SevenEasy, Columbus, OH, USA).

#### 2.5.3. Determination of Moisture Content

The moisture content of the sausage samples was determined using a tabletop infrared moisture analyzer (Sartorius, model MA 35, Göttingen, Germany). The findings were reported as percentages.

#### 2.5.4. Determination of Water Activity

The water activity (a_w_) of the sausage samples was measured using a tabletop dewpoint water activity meter (METER, model Aqualab4TE, Pullman, WA, USA).

#### 2.5.5. Determination of Textural Profile

To determine the textural profile [33], a texture analyzer (Stable Micro Systems, model TA-XT plusC, Godalming, Surrey, UK) was utilized. For this analysis, six cuboid samples (15 × 15 × 15 mm) were thoroughly extracted from the interior of each sausage sample, ensuring the removal of a 3 mm external layer prior to TPA evaluation. Each sample underwent dual compression tests to ascertain attributes such as hardness (measured in grams force), springiness (measured in %), gumminess (measured in grams force), and chewiness (measured in grams force). The equipment settings included the employment of a P/50 probe in compression mode, an auto-10 g trigger type, pre-test, test, and post-test speeds of 2.0 mm/s, 2.0 mm/s, and 5.0 mm/s respectively, along with a strain setting of 50%.

#### 2.5.6. Determination of Lipid Oxidation

##### Extraction of Lipids

To extract the lipids from the sausage samples [34], a 30 g sample was homogenized at 4 °C using a high-shear homogenizer (IKA homogenizer, Model T25 digital ULTRA-TURRAX, Germany), along with 210 mL of chloroform: methanol: distilled water mixture in a 60:120:30 ratio. The homogenizer was set to a speed of 10,000 rpm for a span of 1 min. Subsequently, 60 mL of chloroform was added to dilute the homogenate, which was then re-homogenized under the same conditions, but for a shorter period of 30 s. This dilution and homogenization step was repeated twice. Post homogenization, the mixture was centrifuged using a centrifuge (Sorvall, model RC 5C Plus, Asheville, NC, USA) at 4 °C for 10 min at 5000× *g*. The supernatant was carefully decanted into a separating flask. The chloroform layer was then transferred into a separate 250 mL Erlenmeyer flask and combined with 2–5 g of anhydrous sodium sulfate. After vigorous mixing, the solution was then decanted into a round-bottom flask using a Whatman No. 4 filter paper. Subsequently, the solvent was evaporated at 40 °C with a rotary evaporator (Eyela, Model N-100, Tokyo, Japan), and any remaining solvent was purged with nitrogen. The extracted lipid was then subjected to both TBARS and PV analyses.

##### Determination of Peroxide Value (PV)

The PV was calculated following the methodology described by Low and Ng [35]. A quantity of 1.0 g of the lipid sample was combined with 30 mL of an organic solvent mixture composed of chloroform and acetic acid in a ratio of 2:3. This blend was shaken vigorously and subsequently treated with 0.5 mL of saturated potassium iodide solution. After allowing the mixture to sit in darkness for 1 min, 30 mL of distilled water was introduced, and the combination was agitated once more. A 1% *w*/*v* starch solution, measuring 0.5 mL, was added to the mixture to serve as an indicator. The PV was ascertained by performing a titration on the iodine released from potassium iodide using a standardized 0.01 N sodium thiosulfate solution. The following formula was used to calculate PV and the obtained results were reported as milliequivalents of liberated iodine per kg of lipid:

$$\mathrm{Peroxide}\;\mathrm{value}\;\left(\mathrm{PV}\right)=\left(\frac{V\;\times \;N\;\times 1000}{W}\right)\times 1000\;\mathrm{meq}/\mathrm{kg}$$

where:

V = Volume of saturated potassium iodide solution used in the titration (mL);

N = Normality of saturated potassium iodide solution;

W = Weight of the sample (g).

##### Determination of Thiobarbituric acid Reactive Substances (TBARS)

The TBARS analysis was performed following the procedure outlined by Rajasekaran et al. [36] with some modifications. A quantity of 0.5 g of lipid extraction was combined with 2.5 mL of TBA reagent. The resulting mixture was heated at 95 °C for a duration of 10 min. Following this, the absorbance was measured at 532 nm using a UV-160 spectrophotometer (Shimadzu, Kyoto, Japan). The TBARS value was determined using a standard curve of MDA (0–5 µm), with results reported in mg MDA/kg of oil sample.

### 2.6. Microbiological Analysis

The determination of Total Viable Counts (TVC) and psychrotrophic bacteria counts was carried out in the following manner [37]. A sample of sausage weighing 25 g was blended with 225 mL of sterile Butterfield’s phosphate buffered water for 2 min in a sterile blender jar (Waring Laboratory, model 8010, Torrington, CT, USA), resulting in a sample concentration of 0.1 g/mL. Sterile Butterfield’s phosphate diluent was used to create sequential 10-fold dilutions, achieving sample concentrations ranging from 10^−2^ to 10^−8^ dilutions. The TVC and psychrotrophic counts were then ascertained via the pour plate method, with the plate count agar serving as the medium. These diluted samples were incubated either at 35 °C for 48 h, or at 7 °C for a period of 10 days. The bacterial counts are expressed in terms of log CFU/g. The lactic acid bacteria (LAB) counts were determined by incubating samples on DeMan, Rogosa, and Sharpe agar at 30 °C for 72 h [38]. The resulting counts were expressed in terms of log CFU/g.

### 2.7. Statistical Analysis

All experiments were conducted in triplicate. The results are expressed as the mean ± standard deviation. The data were analyzed using Analysis of Variance (ANOVA), and mean comparisons were performed using Duncan’s multiple range test. Differences were deemed statistically significant if the *p*-value was less than 0.05.

## 3. Results and Discussion

### 3.1. ATF Properties

#### 3.1.1. Proximate Composition

**Figure 1:**
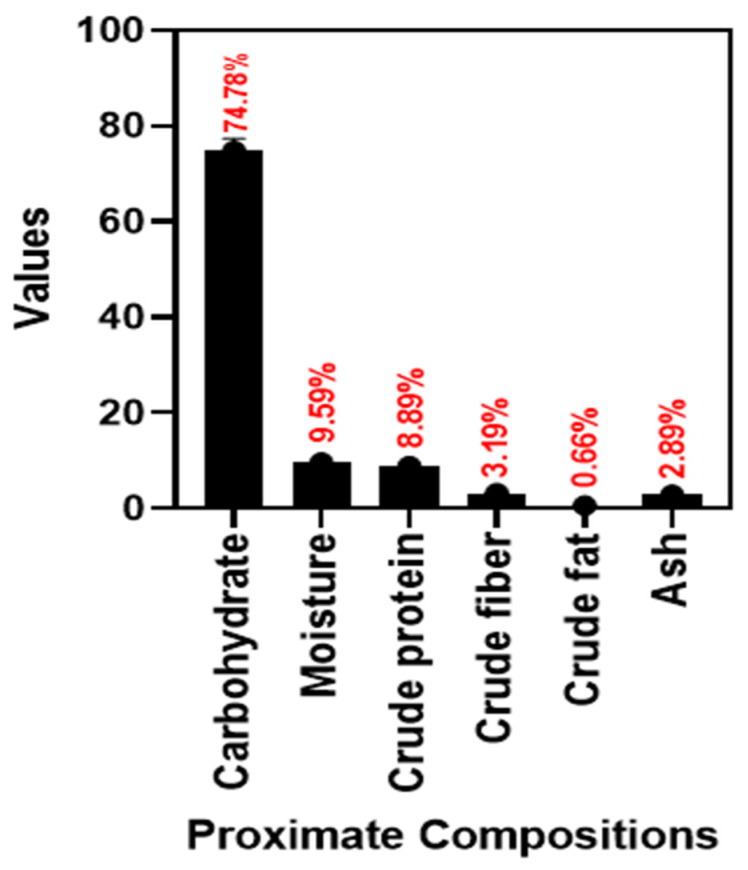
Proximate composition of ATF.

The proximate composition of the arrowroot tuber flour (ATF) produced from arrowroot harvested after 12 months in Figure 1. Notably, there were significant variations in the compositional values corresponding to the tested properties. The carbohydrate content (74.78%) was the highest, followed by moisture content (9.59%), protein content (8.89%), fiber content (3.19%) and ash content (2.89%), in that order. Among all the analyzed components, the fat content (0.66%) was the least. Normally, the proximate composition of AT is greatly varied with the genetic varieties, cultivation area/geographical location, temperature, irrigation, soil nutrition and postharvest processing [39]. Additionally, Soem [27] examined the effects of different cultivation periods (6, 9, and 12 months) on AT and their study determined that cultivation time significantly impacted the total yield of ATF. Among the three time periods, harvesting at the 12-month mark led to the highest percentage increase in ATF yield. Martinescu et al. [40] reported that the proximate composition of ATF is in comparison with rice flour and almond flour and their finding showed that carbohydrate was the predominant component in the ATF. Furthermore, Grosso et al. [41] reported that the vast differences in the levels of carbohydrates and proteins in a particular edible plant source could be attributed to variations in extraction procedures. Plant-based carbohydrates and proteins are good sources of natural materials that are widely used to produce edible coatings [42,43]. Mahajan et al. [44] used arrowroot flour as a thickening agent in ice cream for smooth texture and creaminess, indicating that arrowroot has good pasting properties. This study has also tested the pasting properties of ATF. Shah et al. [45] reported that plant-based polymers that exhibited good pasting properties could serve as a suitable ingredient for developing edible films and coatings. This is in accordance with the present study (see Section 3.1.2).

#### 3.1.2. Pasting Properties

**Figure 2:**
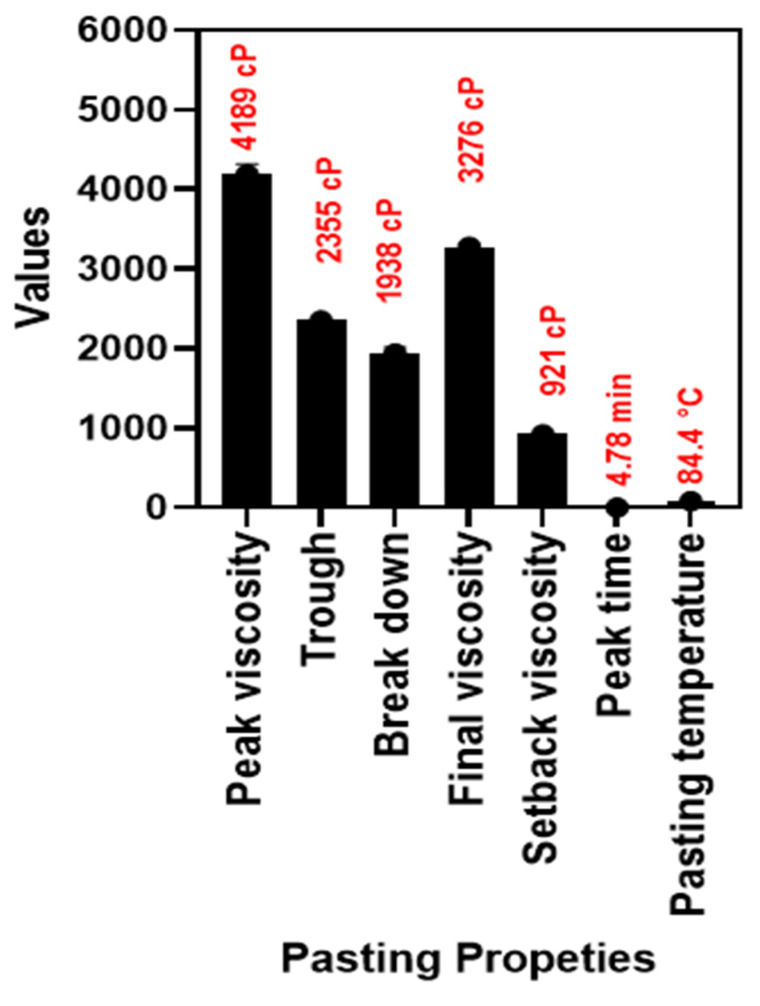
Pasting properties of ATF.

Tubers are an excellent flour source and are an excellent ingredient for food products. The pasting properties are the important characteristics of the flour or starch-based raw materials and the Rapid Visco Amylograph (RVA) is an essential tool for identifying the physical characteristics of flour-based products. Figure 2 illustrates the differences in the RVA properties for arrowroot tuber flour. Overall, the RVA properties of ATF exhibited significant differences in values among each other. Ragaee and Abdel-Aal [46] reported that the differences in the RVA properties of the starch-based flour are mainly dependent on the rate of absorption and swelling properties. Generally, the pasting property of crude ATF is very high as compared with native arrowroot starch (ATF) and this is mainly because of the additional components such as proteins, fats, and fibers present in the ATF and those are missing in the ATS. Higher viscosity values indicate excellent pasting properties. In our study, the peak viscosity of the ATF was notably high, followed by the final viscosity. Other RVA properties, particularly trough and breakdown, were not as high but were within a comparable range. Sholichah et al. [47] reported that ATF tends to have high peak viscosity and low bread down values and its mainly due to the starch content in the ATF entanglement with the other flour components and forming the 3D structural network and thus adversely affects the properties of RVA. This is in accordance with the present study. Zaidul et al. [48] reported that a strong difference between the peak viscosity and final viscosity indicates an amylopectin-rich flour. Among the various viscosity parameters, the setback viscosity was the least. The pasting temperature stood at 84.4 °C, and it took 4.78 min to achieve the complete pasting property of the ATF. This could be due to the fact that ATF flour is rich in amylopectin and is very susceptible to heating as compared with amylose. This is in accordance with the study by Juhász and Salgó [49].

### 3.2. Structural Characterization of ATF-KEO Coating

#### 3.2.1. FTIR

**Figure 3:**
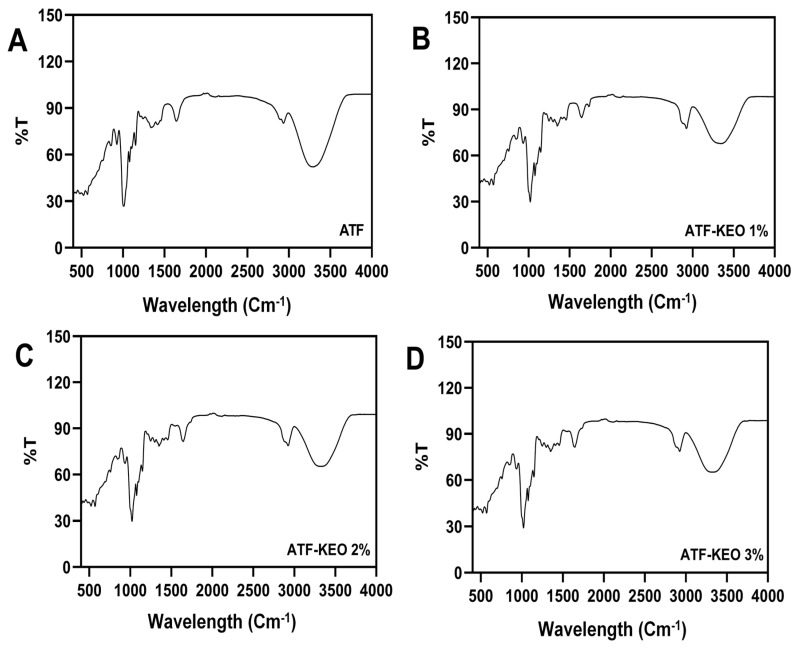
FTIR spectra of edible coating made of ATF incorporated with various concentrations of KEO (**A**–**D**).

FTIR was utilized to identify the functional group of the ATF-based edible coating, which incorporates the KEO at different concentrations. The results are depicted in Figure 3. The FTIR results of the tested material displayed a wide range of bands within the given wavelengths (400–4000 cm^−1^). Most of the peaks in all the samples were observed within the range of 400 to 1700 cm^−1^. There were significant differences in the percentage of transmission between the control samples and the samples with added KEO. The control samples exhibited FTIR peaks at 3341.27, 2924.21, 1644.06, 1455.49, 1350.24, 1248.12, 1147.10, 1077.82, 1020.01, 933.11, 847.86, 757.78, 570.81, and 521.88 cm^−1^. On the other hand, the samples with added KEO showed average FTIR peaks at 3313.80, 2927.28, 1636.56, 1372.19, 1277.64, 1148.69, 1077.47, 1016.66, 930.95, 849.58, 570.61, 522.27, and 416.97 cm^−1^. The addition of KEO altered the FTIR peaks in comparison with the control samples. Furthermore, an increase in KEO concentrations slightly increased the percentage of transmission, but the placement of the FTIR peaks remained consistent despite the variations in KEO concentrations. Overall, the analysis of the coating samples revealed that the higher percentage (>60%) of FTIR peak transmission fell between 1000 and 3000 cm^−1^, with extended band stretching observed in the samples with added KEO. Bands observed in the range of 3400–3250 cm^−1^ represent the N-H stretch, indicative of amines and amides. Tanavar et al. [50] discovered that biopolymers, rich in carbohydrates and protein fragments, often overlap with the stretching of O-H, NH_2_, and secondary NH amides. Alavi et al. [51] reported that finding a band in the wavelength range of approximately 3000 cm^−1^ could be associated with the water molecules in the coating samples, as this range represents the O-H groups. The FTIR bands absorbed by the edible coating between 1600–1699 cm^−1^, 1400–1499 cm^−1^, and 1300–1399 cm^−1^ represent the overlapping absorption of C=O stretching, C-C stretching, and C-H stretching [52]. This is consistent with our study. The absorbent peak was noticed at 1248 cm^−1^ (C-O stretching) in the control sample but was absent in the samples with added KEO. Yashaswini and Iyer [53] found similar results in a chitosan-based edible film incorporated with turmeric essential oil. The band situated between 1000–1100 cm^−1^ in the coating materials represents the O-H group, corresponding to glycerol, primarily used as a plasticizer [54]. This study found that the highest amplitude of the aromatic group (C-H stretching) was present in the coating samples with incorporated KEO. Ramadhan and Iftitah [55] tested the FTIR spectra of the KEO, and their study identified major absorption peaks at 2922 cm^−1^ (C-H stretching), 1710–1665 cm^−1^ (C=O stretching), and 1300–1379 cm^−1^ (C-C stretching), representing aromatic and aldehyde functional groups. This study aligns with their findings, as the addition of KEO in the edible coating resulted in extended absorbent peaks compared to the control coating material lacking KEO.

#### 3.2.2. Microstructural Observations

**Figure 4:**
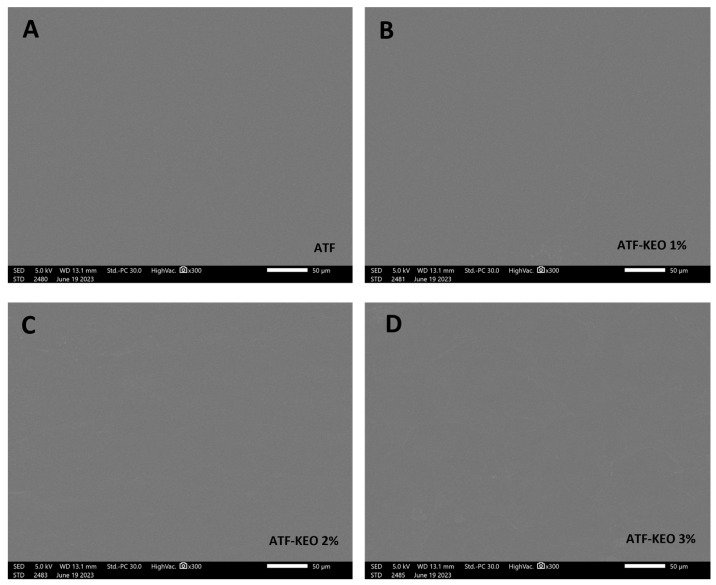
Microstructural changes of edible coating made of ATF that incorporated with various concentrations of KEO (**A**–**D**).

The microstructural observation on the surface of ATF-based edible coating that incorporates different concentrations of KEO is shown in Figure 4. Overall, there are no remarkable microstructural changes observed in all the tested samples. This observation indicates that the mixture of ATF flour, glycerol, and tween 80 produced a smooth homogeneous continuous surface coating without any cracks and bumps on the surface coating. This is in accordance with the study of Ren et al. [56], who reported that such findings on the microstructure are mainly attributed to the good homogenous mixture, which is mainly attributed to the potency of the interaction and compatibility of polymer and plasticizer. Matta Fakhouri et al. [57] observed the microstructure of the biopolymer that was composed of arrowroot starch, and their findings showed that biopolymer made of arrowroot base given the colorless, odorless, and smooth surface with the organized polymer matrix. Similarly, the KEO-added coating samples also exhibited smooth surfaces like the control; however, there were some minor changes in the microstructural surface upon the increased concentration of KEO. However, these changes did not induce any severe agglomerates, cracks and holes on the surface. Gomide et al. [58] reported that the slight changes in the surface microstructure of edible polymers are mainly attributed to the interactions between the polymers, plasticizers and added additives through Van der Waals force and hydrogen bonding. In this case, the lower concentration of KEO did not induce any internal interactions, and however, when the concentrations gradually increased, might have increased some intermolecular attractions in the polymer matrix and thus affected the surface smoothness slightly. However, due to the better compatibility of glycerol and polysorbate with the ATF, the morphological changes in the KEO-added samples were still minimal and exhibited better morphology. Abdillah and Charles [18] reported that arrowroot-based edible polymers are excellent components for producing an edible film or edible coating as they are transparent, odorless and exhibit a very smooth morphology. Nogueira et al. [16] reported that the arrowroot flour/starch-based biopolymer displayed a uniform surface, accompanied by a highly interconnected network structure, which is attributed to its enhanced water resistance capability. As a result, this film emerges as a prime candidate for edible coatings applied to food products, more so for semi-prepared commodities intended for subsequent cooking or direct ingestion with the item.

### 3.3. Impact of ATF-KEO Coating on Pork Sausage Qualities

#### 3.3.1. Physiochemical Qualities

**Figure 5:**
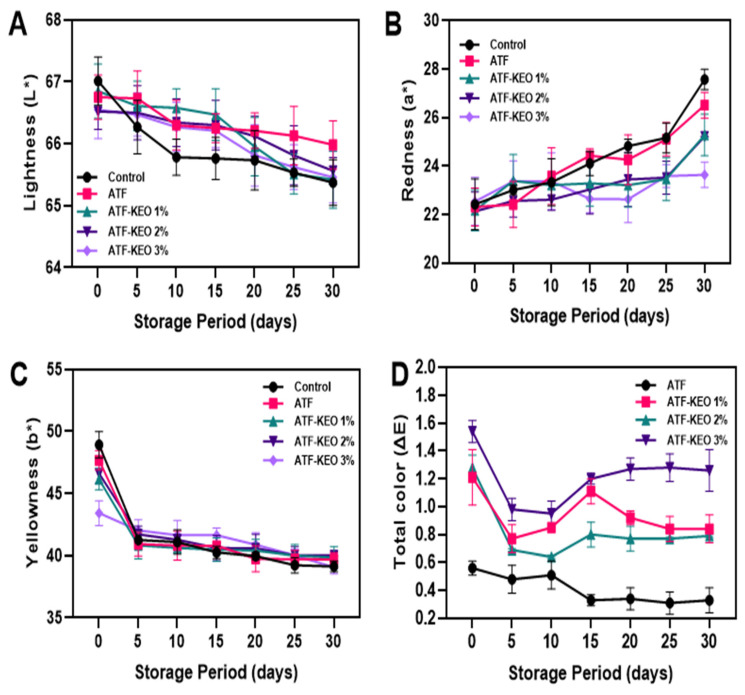
Changes in color characteristics (lightness (**A**), redness (**B**), yellowness (**C**) and total color (**D**) of pork sausages with edible coating made of ATF-KEO at various concentrations and stored under prolonged refrigerated conditions.

The alterations in color characteristics such as L*, a*, b* and total color (ΔE) values in pork sausages, coated with various concentrations of ATF-KEO, are demonstrated in Figure 5. A significant change in the color quality of pork sausages was observed across all samples. The L* and b* values consistently decreased, whereas the a* values increased throughout the storage period (Figure 5A–C). Figure 5D illustrates the alterations in the numerically calculated ΔE values of the pork sausages during storage. The results demonstrate a mixed trend in ΔE changes, despite the variations in the samples. Among the ATF samples, those coated with ATF-KEO 3% exhibited slightly higher ΔE values compared to the others. In contrast, the ATF-KEO 2% samples displayed marginally lower ΔE values. Samples of ATF without KEO maintained the lowest ΔE values in comparison to the others. Moreover, minimal changes in ΔE were observed after 10 days of storage, and subsequently, the prolonged storage period did not significantly impact the values. Luong et al. [59] reported that continuous storage under refrigeration could decrease the L* values in pork sausages. While the extended storage period significantly influenced the color quality of the sausages, no marked differences were noted between the control and coating variations. The results indicated that both the control and ATF-coated samples retained their color characteristics marginally better than the ATF-KEO-coated samples. This suggests that the incorporation of KEO in the sample had a slight impact on the color characteristics. Catarino et al. [60] highlighted that the color of food is determined by various factors. These include the type and concentration of pigments, the levels of water and fats, and the presence of other minor constituents such as essential oils.

**Figure 6:**
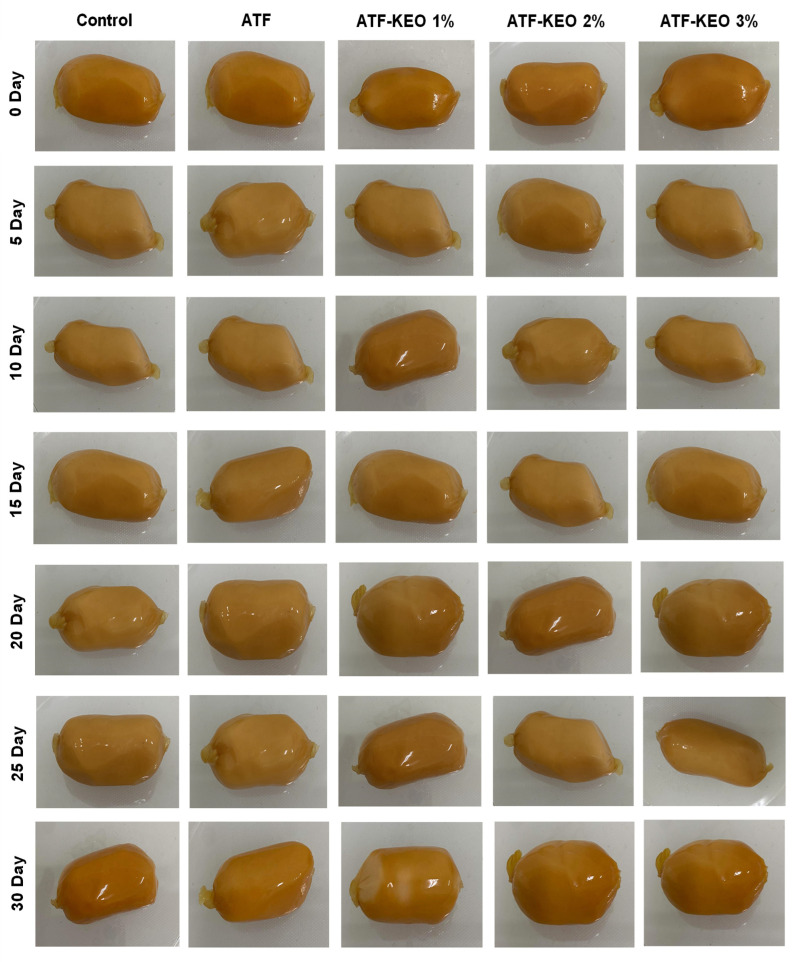
Changes in the appearance of pork sausages with edible coating made of ATF-KEO at various concentrations and stored under prolonged refrigerated conditions.

**Figure 7:**
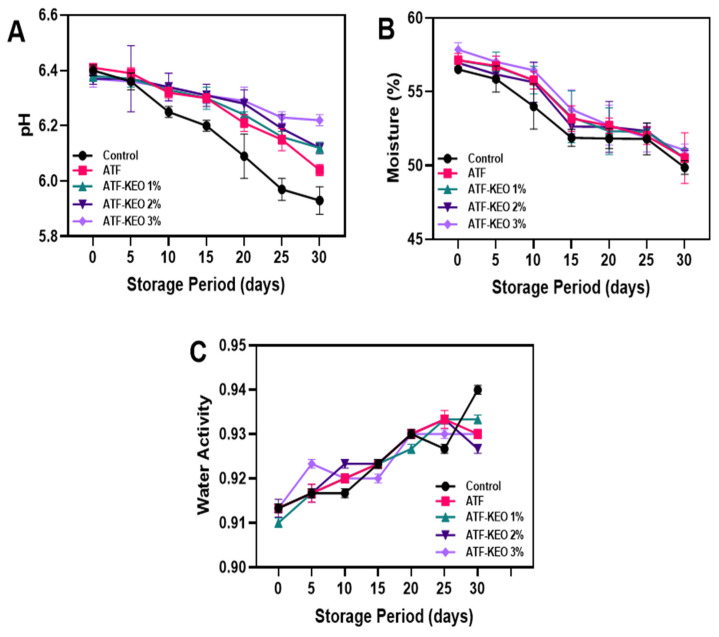
Changes in pH (**A**), moisture (**B**) and water activity (**C**) of pork sausages with edible coating made of ATF-KEO at various concentrations and stored under prolonged refrigerated conditions.

Additionally, the appearance of edible-coated sausages is shown in Figure 6. The observation shows that there are not many changes in the overall appearance of the sausage samples. The changes in pH of edible pork sausage coated with ATF-KEO at various concentrations are displayed in Figure 7A. Over the storage period, there was a gradual decrease in the pH of all samples, although the changes were not greatly varied. Generally, a change in pH accompanies the process of acidification, which is commonly observed during the storage of meat products. This acidification is primarily triggered by the production of acid from microbial metabolism [60]. Among the samples, the control ones experienced a greater decrease in pH, followed by those with ATF coating. Ruiz-Capillas et al. [61] reported that the decrease in pH of pork sausages was primarily attributed to the accumulation of lactic acid, resulting from microbial activity, particularly from lactic acid bacteria. The pH of the samples with ATF-KEO coatings did not significantly differ, regardless of the varying concentrations. Venkatachalam and Lekjing [62] found that an increase in pH in pork meat products was caused by microbial enzymes, and the inclusion of EO in the edible coating significantly reduced this occurrence. Utami Hatmi et al. [19] conducted tests on beef sausages with an edible coating enriched with KEO. Their study revealed that the application of KEO significantly managed the pH fluctuations in the samples by suppressing acid-producing bacteria. The moisture content of pork sausages tends to decrease gradually with extended storage time, as depicted in Figure 7B. Liu et al. [63] reported that the moisture content in pork sausages commonly tends to decrease over time under refrigerated conditions. Papadima and Bloukas [64] also observed a similar finding in the traditional Greek sausages. Control samples, which had no coating, lost a significant amount of moisture. This is in accordance with Kang et al. [65]. In contrast, both ATF and ATF-KEO demonstrated better control in retaining the moisture content. Among the sample types, the ATF-KEO coated samples retained more moisture, and the samples with higher KEO concentrations were the most effective at controlling moisture loss compared to the others. In general, edible coatings with essential oils decrease moisture loss in pork sausages by forming a protective barrier to prevent evaporation [66], and furthermore, the hydrophobic properties of some oils further limit moisture loss by repelling water. On the other hand, the water activity (a_w_) of the pork sausage also tended to slightly increase (Figure 7C). However, these differences were not significant, regardless of the sample type. Generally, pork meat-based products fall within the range of 0.90–0.99, a range that is conducive for most pathogenic bacteria to proliferate more rapidly [67]. The a_w_ of the pork sausage in our study indicates its susceptibility to spoilage due to bacterial and fungal growth. Nevertheless, microbial results suggest that the application of ATF-KEO can control microbial growth, despite the moisture and a_w_ range being favorable for such growth.

#### 3.3.2. Textural Profile

**Figure 8:**
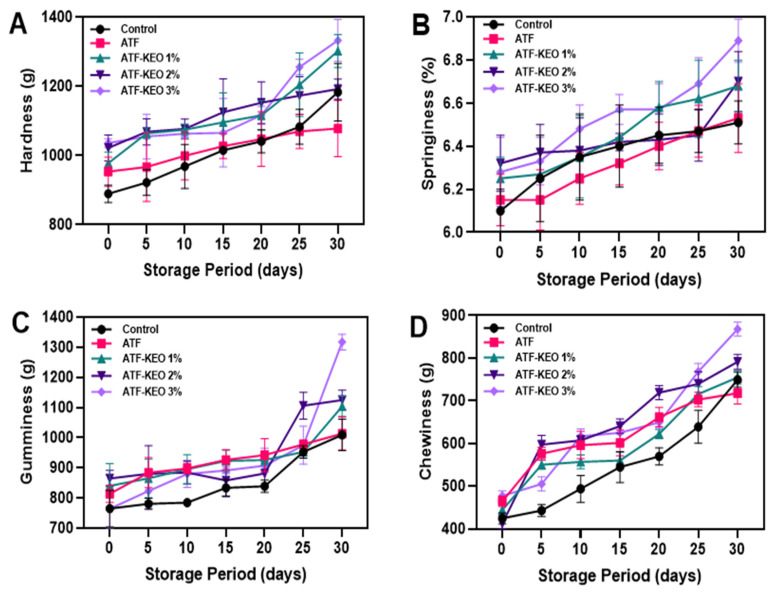
Changes in hardness (**A**), springiness (**B**), gumminess (**C**) and chewiness (**D**) of pork sausages with edible coating made of ATF-KEO at various concentrations and stored under prolonged refrigerated conditions.

The texture profile is a critical measure that demonstrates a sausage’s ability to retain water and fat and indicates its stability as an emulsified meat product [68] and is heavily affected by factors such as the type and concentration of salt and the level of dissociated ions [69]. The textural properties (hardness, springiness, gumminess, and chewiness) of pork sausages coated with ATF-KEO at various concentrations were examined and the results are depicted in Figure 8A–D. It was found that prolonged storage gradually influenced these properties. Initially, there were no significant differences in the hardness values among the sausages despite sample variations. However, with extended storage time, sausages coated with ATF-KEO consistently retained higher hardness values compared to other samples (Figure 8A). Storage time and sample variations had minimal impact on the springiness values (Figure 8B). Across all samples, a non-significant improvement in springiness was observed throughout storage. Springiness, which quantifies a sample’s ability to regain its original height following deformation (such as a first bite), is often considered an important indicator of protein richness and fluffiness in sausages [70]. Minimal changes in springiness suggest the overall palatability of the sausages remained constant. During the storage period, gumminess and chewiness values steadily increased across all samples. Control samples exhibited the lowest values, followed by the ATF and ATF-KEO samples (Figure 8C,D). Jokanovic et al. [71] noted that drying during storage progressively impacts textural properties, negatively affecting binding and rheological attributes and increasing chewiness, a change that correlates with alterations in the sausage’s moisture content. On the other hand, the gumminess values consistently recorded higher values than chewiness. Overall, sausages coated with ATF-KEO managed to maintain their textural properties well. Among different KEO concentrations, the 3% concentration performed marginally better than other concentrations. For refrigerated storage, sausages could benefit from increased firmness, which aids in moisture and fat retention, thereby extending shelf-life. However, the impact can vary depending on the specific product formulation and the application of antimicrobial coatings. Ashaolu et al. [72] reported that the lipolytic and catalytic activities of lipase and catalase enzymes, predominantly produced by spoilage microorganisms like *L. plantarum*, *L. sakei*, and *S. warneri* are primarily responsible for adverse changes in sausage texture. Alizadeh Behbahani et al. [73] found that the incorporation of essential oils into edible coatings could delay the degradation of collagen and myofibrillar proteins in red meat-based products. This delay is achieved by controlling the activity of microorganisms and endogenous enzymes, particularly cathepsins, collagenases, and calpains. Tabanelli et al. [74] reported that a decrease in microbial growth had significantly enhanced the texture profile of the sausage.

#### 3.3.3. Lipid Oxidation

**Figure 9:**
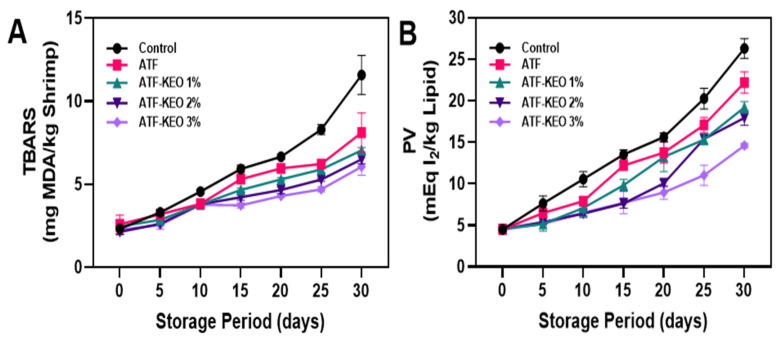
Changes in TBARS (**A**) and PV (**B**) of pork sausages with edible coating made of ATF-KEO at various concentrations and stored under prolonged refrigerated conditions.

Lipid oxidation in edible pork sausages, coated with ATF that incorporate various concentrations of KEO, is depicted in Figure 9A,B. Lipid oxidation is a key parameter used to identify quality deterioration in meat-based products. It is typically measured in food products using TBARS and PV [75]. Thiobarbituric active reactive substances (TBARS) are one of the secondary oxidation products produced by the breakdown of oxidized PUFA, which is a widely used assay to measure lipid peroxidation end-product malondialdehyde [76]. The application of an ATF coating and varying concentrations of KEO significantly controlled the TBARS levels in the pork sausages (Figure 9A). Results indicate that while the control samples continuously increased in TBARS values throughout storage, the treated samples exhibited no differences initially (0–5 days). This suggests that the onset of lipid oxidation was initially controlled by refrigeration and packaging, while the treatment with ATF and ATF-KEO played a key role in suppressing TBARS values thereafter. Charles et al. [77] studied the encapsulation ability of arrowroot starch (ATS) and their study found that encapsulation of tuna fish oil with ATS significantly controlled the TBARS values as compared with the maltodextrin. Although ATF shows a better effect compared with the control, overall, the ATF-KEO samples performed better. Furthermore, increased KEO concentrations significantly extended the TBARS-controlling effect. Klangpetch et al. [78] studied the anti-oxidation effect of KEO on the chicken drumettes, and their study found that the application of KEO had decreased by around 40% of lipid oxidation in the sample in comparison with the control. Similar to TBARS, PV also makes a critical contribution to lipid oxidation in food samples [79]. PV is associated with an increased formation of hydroperoxides, which are primary oxidation products. These reflect oxidative deterioration in high-fat food products [80]. Furthermore, a high PV value in food can expedite the onset of rancidity, leading to unwanted flavors. The results showed that the PV values tended to increase continuously in the pork sausages during the extended storage, despite the sample variations (Figure 9B). Utami Hatmi et al. [19] examined the effectiveness of an ATF coating in controlling PV. Their study found that, compared to coatings based on cassava and canna edulis flour, the ATF coating was notably more successful at managing PV levels. However, this study also found that the PV values of the ATF-KEO coated samples were also significantly lower when compared with ATF and the control samples. Boran et al. [81] reported that 8–10 meq/kg is the acceptable limit for PV in oil-based food for human consumption. This study showed that ATF-KEO coatings are in accordance with their recommended range. Alparslan et al. [82] proposed that the effectiveness of essential oils, especially from the citrus family, in inhibiting lipid oxidation when used in edible coatings is largely due to their antioxidant properties. They further reported that essential oils from orange peel and kaffir lime are among the most potent sources of such antioxidant power. Abirami et al. [83] reported that the antioxidant potency of kaffir lime and its derived products primarily stems from their high content of phenolics and flavonoids.

#### 3.3.4. Microbiological Growth

**Figure 10:**
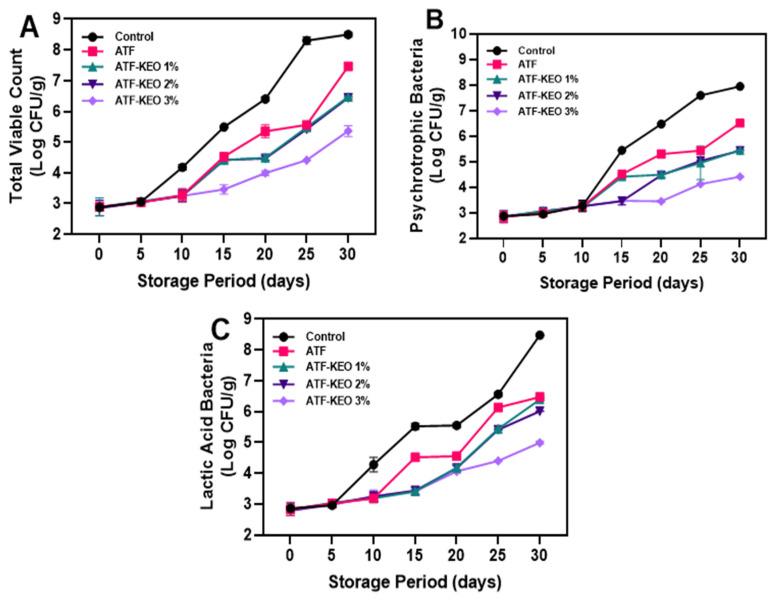
Changes in microbial growth (Total viable count (**A**), Psychrotrophic Bacteria (**B**) and Lactic acid bacteria (**C**) of pork sausages with edible coating made of ATF-KEO at various concentrations and stored under prolonged refrigerated conditions.

Microbial growth on the pork sausages with edible coatings of ATF and KEO at different concentrations is illustrated in Figure 10A–C. Overall, all tested parameters exhibited a continuous increase in microbial growth over the storage period; however, the ATF-KEO sample variations were able to the microbial growth slightly throughout the storage period. Generally, the application of plant-based extracts incorporated with the edible coatings could be able to substantially control the growth of spoilage microorganisms [84]. Essential oils, recognized as secondary metabolites, possess robust antimicrobial properties. The capability of these oils to inhibit pathogenic and spoilage bacteria, however, is contingent upon their specific chemical compositions (alcohol, aldehyde, esters, ethers, and methoxy derivates) [85]. Additionally, the performance of individual compounds within the essential oils appears to fluctuate according to the developmental stages of the implicated organisms [86]. Figure 10A depicts the total viable count (TVC) of the pork sausages. The results showed that the addition of KEO to the edible ATF coating significantly helped to reduce the TVC growth rate. The effect of KEO was dose dependent; increased KEO concentrations significantly reduced the TVC level. However, the low to medium KEO concentrations did not significantly differ from each other in controlling the TVC level in the samples. Sausages treated with 3% KEO exhibited superior control against the increase in TVC level compared to the other tested samples. Notably, the control samples exhibited a heightened level of TVC throughout the storage. Sreepian et al. [87] reported that KEO rich in limonene and β-pinene are the predominant compounds that exhibit strong antimicrobial activities. Utami et al. [88] investigated the impact of KEO on the TVC level in stored beef sausages and their findings indicated that incorporating a higher concentration of KEO significantly mitigated the growth of TVC, showcasing a more potent effect than the lower concentrations and the control group. Similarly, the growth of psychrotrophic bacteria on the pork sausages was significantly controlled by the ATF-KEO coatings compared to the samples that included ATF-coated and control samples. However, the differences between the ATF-KEO samples with low concentrations were not significant. Figure 10B indicates that at the initial storage period, the psychrotrophic bacterial growth in the samples was within an acceptable range and did not significantly differ among the samples. However, when the storage period exceeded 10 days, growth rapidly escalated, with the control showing the highest growth, followed by the ATF and ATF-KEO samples. Gedikoğlu [89] reported that the addition of essential oil in the coating emulsion to exhibit the synergistic or antagonistic effect is mainly dependent on the concentration of the compound used and the releasing power of the coating emulsion during the prolonged storage. During aerobic storage at chilled temperatures, *Pseudomonas* spp. often predominates, while lactic acid bacteria tend to dominate under refrigerated storage conditions [85]. The lactic acid bacterial growth in the pork sausages tended to increase over the storage period (Figure 10C) regardless of the variations in the samples. The control and ATF coating did not significantly differ, but the ATF-KEO coating with higher concentrations showed slight control towards the end of storage. Initially, there were not many differences in the values. Although the microbial results continuously increased, the values remained within the acceptable range for most of the storage period. In accordance with the Department of Medical Sciences, Thailand [90], food products that are either cooked or uncooked are considered inappropriate for consumption when the microbial growth is in the more than acceptable range (>log 6 CFU/g). The present results indicate that pork sausages coated with ATF and higher KEO concentrations are safe for consumption up to 20 days of storage.

## 4. Conclusions

The present study investigated the use of arrowroot tuber flour (ATF), which is high in carbohydrates, combined with KEO as a coating for pork sausages. The research revealed that ATF demonstrated notable variations in RVA properties, underscoring its multifaceted starch behavior. Upon integration with KEO, shifts in FTIR peak values were observed, indicating potential alterations in the properties of the product. While KEO introduced minor modifications in the color attributes and microstructure of the sausage, it did not significantly impact the pH, moisture content, or water activity. Importantly, the ATF-KEO coatings played a pivotal role in preserving the textural integrity of the sausage, particularly its hardness, throughout the storage period. The study uncovered the efficacy of KEO in mitigating lipid oxidation and microbial proliferation, resulting in a significant decrease in the total viable count. In conclusion, coatings formulated with ATF and 2–3% KEO could enhance the hardness, color stability, and microbial safety of pork sausages, highlighting the importance of continued exploration to broaden their utilization in food preservation.

## Data Availability

The data used to support the findings of this study can be made available by the corresponding author upon request.
